# Do South Korean adults have insufficient levels of physical activity? Domain patterns and associations with self-rated health

**DOI:** 10.1371/journal.pone.0352382

**Published:** 2026-06-22

**Authors:** Kyoung Ae Kong

**Affiliations:** Department of Preventive Medicine, College of Medicine, Ewha Womans University, Seoul, Korea; Universiti Malaya, MALAYSIA

## Abstract

**Objectives:**

The study aimed to evaluate domain-specific physical activity (PA) patterns in South Korea, where the prevalence of insufficient PA is high, and assess their association with self-rated health (SRH).

**Methods:**

Data from 32,005 South Korean adults aged 20–79 from the Korean National Health and Nutrition Examination Survey (2016–2021) were analyzed. Domain-specific PA (leisure-time (LTPA), transportation-related (TPA), and work-related (OPA)) were measured using the Global Physical Activity Questionnaire. PA was categorized into <10, 10–149, and ≥150 min/week, labeled as “inactive,” “insufficient,” and “meeting PA guidelines (PAG).” Each individual’s primary PA domain was defined by the domain with the largest proportion of their total PA time. Logistic regression was used to examine associations between PA patterns and good SRH.

**Results:**

In total, 46.3% met PAG, while 30.8% were inactive. TPA was the primary domain for 59.0% of non-inactive individuals. This emphasis on TPA was pronounced among women, the elderly, and those with insufficient PA. Only 8.7% had ≥ 10 minutes of continuous OPA per week, and 19% met PAG through LTPA alone. LTPA showed a clear positive dose-response relationship with good SRH. TPA showed a weak positive association, while OPA showed an inverse association.

**Conclusions:**

The high prevalence of insufficient PA among Korean adults is likely primarily attributed to low levels of OPA. TPA is the primary PA domain. Only LTPA showed clear health benefits regarding SRH, but a large proportion did not meet PAG through LTPA. Monitoring domain-specific PA patterns is needed for developing effective PA promotion strategies in Korea.

## Introduction

Regular physical activity (PA) has health benefits such as the lower risk of all-cause and cardiovascular diseases mortality, incidence of cardiovascular disease, hypertension, type 2 diabetes, adverse lipid profile, breast and colon cancer, the improved mental health, and the maintenance of healthy weight [1,2]. WHO recommends at least 150–300 minutes of moderate-intensity aerobic PA, or at least 75–150 minutes of vigorous-intensity aerobic PA, or an equivalent combination of moderate- and vigorous-intensity activity per week for adults aged 18–64 years and older adults aged over 65 [1].

PA is defined as any bodily movement generated by skeletal muscles that demands energy expenditure and can occur at various intensities, involving tasks at work, domestic chores, transport, and sporting and recreational activities. Consistent with this definition, WHO derives its global estimates of PA levels based on self-reported data obtained through questionnaires covering various activities at work or in the household, during transportation, and in leisure-time, using tools such as the Global Physical Activity Questionnaire (GPAQ) and the International Physical Activity Questionnaire [3].

However, the health effects of PA may vary depending on the context in which it occurs. Leisure-time physical activity (LTPA, e.g., sports, fitness, and recreational activities) is generally reported to have clear health benefits and has been the primary basis for physical activity guidelines (PAG). By contrast, recent studies have shown that occupational physical activity (OPA, e.g., paid or voluntary work, including household chores) may increase rather than reduce the risk of cerebrovascular disease and all-cause mortality [4–6]. Active transportation is considered an effective strategy to increase PA levels for health benefits [7–9], but the relationship between transportation-related PA (TPA, e.g., walking or cycling for travel) and an increase in LTPA, as well as the health benefits based on leisure activity itself rather than the amount of PA, remains unclear [9,10].

South Korea has an exceptionally high prevalence of insufficient PA among adults, with the age-standardized prevalence among adults aged ≥18 years estimated at 56.5% in 2021, compared with the global estimate of 30.8%, according to the WHO Global Health Observatory [3]. Based on these WHO estimates, South Korea ranked within the top 10 among 195 countries in each year from 2016 to 2021 for this indicator. The 5^th^ National Health Plan (HP2030) of Korea uses the proportion of adults meeting aerobic PAG as a key indicator, drawing on the total aerobic PA time calculated from the GPAQ data in the Korean National Health and Nutrition Examination Survey (KNHANES), which sums PA bouts across different domains [11]. However, domain-specific PA patterns among Korean adults have not been monitored or assessed using these data.

Therefore, this study aimed to evaluate domain-specific PA patterns, including PA levels and the prevalence of meeting PAG based on these activities, among South Korean adults to explain the high prevalence of insufficient PA, and to assess their association with good self-rated health (SRH) as a proxy for overall health outcomes.

## Methods

### Data source and study subjects

The data source for this study was the 2016–2021 KNHANES. KNHANES is an ongoing national survey conducted annually by the Korea Disease Control and Prevention Agency to monitor the health behaviors, health status, and nutritional status of the Korean population [12,13]. The survey includes health interviews, health examinations, and nutrition surveys, and it samples the civilian, non-institutionalized population in South Korea using a complex, multistage, clustered probability sampling design. In the 2016–2021 KNHANES, each annual survey included approximately 10,000 eligible individuals selected from sampled geographic units and households, with participation rates averaging 75–78%. For all KNHANES surveys, written informed consent is obtained from all participants, and anonymized, de-identified data are made publicly available through the KNHANES website for research purposes.

Of the 35,688 South Korean adults aged 20–79 who participated in the health surveys during 2016–2021, 3,388 subjects who did not respond to the questions regarding aerobic PA were excluded. A further 295 subjects were excluded because of missing or invalid responses or measurements regarding educational level, household income, occupation, chronic disease history, PA limitations, SRH status, and body mass index (BMI). In total, 32,005 subjects were included in the analysis. This study was determined to be exempt from review by the Institutional Review Board of Seoul Hospital of Ewha Womans University Medical Center (IRB No: SEUMC 2024-08-063) as it involved the analysis of publicly available anonymized data.

### Study variables

#### Physical activity measurement and derived variables.

In KNHANES 2016–2021, PA levels were assessed using the GPAQ developed by WHO [14]. The questionnaire comprised questions regarding the number of days per week and the typical daily duration spent on moderate- or vigorous-intensity activities lasting at least 10 minutes during paid or voluntary work including household chores (OPA), during travel to and from places (TPA), and in sports, fitness, and recreational activities (LTPA). Intensity classification was not applied to TPA, which was considered moderate-intensity. Intensity-specific activity times were calculated by multiplying the number of days per week by the daily PA time spent in each domain. Domain-specific PA time was estimated by summing the moderate and vigorous activity times, which were doubled to account for its equivalent in moderate-intensity time, for each domain. Total PA time was estimated by summing the times across all three domains. In this study, all PA times refer to moderate-intensity equivalent times.

For analysis, the total and domain-specific PA times were divided into <10, 10–149, 150–299, 300–599, 600–899, and ≥900 min/week. These were further classified into three levels: < 10, 10–149, and ≥150 min/week, and labeled as “inactive,” “insufficient,” and “meeting PAG,” respectively. To differentiate PA patterns, among subjects who were not inactive, we classified the primary PA domain for each individual based on the domain accounting for the largest proportion of their total PA time, categorizing it as leisure/sports, transportation, work, or “else.” In cases where ≥2 domains accounted for the largest proportion, the individual was categorized as “else.” We also classified the contribution of each domain-specific PA to an individual’s total PA as 0, 1–24, 25–49, 50–74, and 75–100%.

#### Self-rated health.

SRH was used as a surrogate for overall health outcomes to evaluate the association with PA patterns. In KNHANES, SRH is measured using a single question reflecting respondents’ overall subjective evaluation of their health. The question asks, “In general, would you say your health is?” with five response options ranging from “very good” to “very poor,” with “very good” and “good” grouped as “good SRH.”

#### Sociodemographic and health-related covariates.

Sociodemographic and health-related covariates were obtained from the KNHANES health interview and questionnaire data, except for BMI, which was calculated from height and weight measured in the health examination. These covariates comprised gender, age (in 10-year intervals), educational level (≤middle school graduate, high school graduate, or ≥college graduate), household income quartile group, occupation (manual, non-manual, or others), residential area (metropolitan cities, cities, or rural areas), BMI (<17.9, 18–22.9, 23–24.9, 25–29.9, or ≥30 kg/m^2^), the number of chronic diseases, and PA limitations. The number of chronic diseases was defined as the number of illnesses diagnosed by a physician among cancer, stroke, myocardial infarction or angina, hypertension, dyslipidemia, diabetes, thyroid diseases, chronic liver diseases, kidney diseases, asthma, depression, osteoarthritis or rheumatic arthritis, and gout. Chronic mental health conditions other than depression were not included in this count because depression was the only chronic mental health condition consistently assessed among adults in the KNHANES chronic disease variables during the study period. Limitations in PA were defined as having to stay in bed due to health problems on the survey day or being unable to climb stairs in the past week; having current fractures, joint or other injuries, arthritis, rheumatism, back/neck problems, knee/leg pain, stroke, dementia, mental retardation, vision/hearing problems, or dizziness that restrict daily/social activities; spending almost the entire day in bed because of illness or injury in the past month; or experiencing discomfort due to illness, accidents, or poisoning in the previous two weeks.

### Statistical analysis

We first described the sociodemographic and health-related characteristics of the study subjects overall and according to total PA level. We then estimated the prevalence of inactivity, insufficient PA, and meeting PAG among Korean adults during 2016–2021, stratified by gender and age group (20–34, 35–49, 50–64, and 65–79 years). The distributions of total and domain-specific PA time were also estimated by gender and age group. Among those who were not inactive, we estimated the distribution of the primary PA domain and the contribution of each domain-specific PA to total PA time.

To assess the health effects of the domain-specific PA, we explored the association between good SRH and total PA time, domain-specific time, primary domain, and contribution of each domain-specific PA to total PA time using logistic regression analysis while considering complex survey design. We included gender, 10-year age groups, education level, income quartile, occupation category, residential area, BMI group, number of chronic diseases, and limitations in PA in all models. Additionally, the models for the primary PA domain and the contribution of each domain-specific PA included total PA time as a covariate, while the model for the domain-specific PA time included all three domain times together for mutual adjustment. As good SRH was analyzed as a binary outcome and all explanatory variables were entered as categorical variables, logistic regression was considered appropriate for the analyses, and assumptions related to linearity in the logit for continuous predictors were not applicable. In accordance with the complex sampling design, all analyses were performed considering sampling weight, which accounts for the unequal probabilities of selection, nonresponse, gender and age distribution of the target population, and conducted with SAS statistical software (version 9.4; SAS Institute; Cary, NC).

## Results

### Sociodemographic and health-related characteristics of study subjects

In total, the number of individuals meeting the PAG, those with insufficient PA, and those considered physically inactive were 13,961, 7,512, and 10,532, respectively (Table 1). Individuals meeting the PAG were more likely to be younger, male, metropolitan residents, with higher socioeconomic status, and no chronic disease or history of PA limitations.

**Table 1:** Sociodemographic and health-related characteristics of study subjects by total physical activity level groups.

		Total (n = 32,005)		Not meeting PAG		Meeting PAG (n = 13,961)
				Inactivity (n = 10,532)	Insufficiency (n = 7,512)	
		N	col%	col%	col%	col%
Gender	Men	14129	50.2	49.6	44.1	53.5
	Women	17876	49.8	50.4	55.9	46.5
Age (year)	20–29	3917	17.3	9.8	15.4	23.3
	30–39	4980	18.2	17.1	17.5	19.4
	40–49	6094	20.7	20.8	20.7	20.7
	50–59	6413	20.8	23.1	21.8	18.7
	60–69	6191	14.5	17.4	14.8	12.5
	70–79	4410	8.4	11.8	9.7	5.4
Educational level	≥College	12641	44.0	36.9	43.7	48.8
	High school	10703	36.1	35.3	34.4	37.5
	<Middle school	8661	19.9	27.8	21.9	13.7
Household income quartile	High	8126	25.1	22.2	24.6	27.2
	Mid-high	8069	25.3	24.9	24.9	25.9
	Mid-low	8029	25.1	26.1	25.3	24.2
	Low	7781	24.5	26.8	25.1	22.7
Occupation	Non-manual	8345	29.3	26.0	28.5	31.8
	Manual	11738	36.7	41.8	33.6	34.8
	Others	11922	34.1	32.2	37.9	33.4
Residential area	Rural areas	5991	14.7	20.8	13.4	11.4
	Cities	11784	39.6	40.6	39.8	38.9
	Metropolitan cities	14230	45.6	38.6	46.9	49.7
BMI (kg/m^2^)	<17.9	754	2.5	2.5	2.7	2.4
	18–22.9	12421	38.7	37.0	41.2	38.6
	23–24.9	7343	22.5	22.8	21.8	22.7
	25–29.9	9584	30.0	31.2	28.3	29.9
	≥30	1903	6.3	6.5	5.9	6.3
Number of	0	16795	58.8	53.1	58.6	62.7
chronic diseases	1	7109	21.1	22.4	20.6	20.5
	2	4243	11.0	12.5	10.9	10.0
	≥3	3858	9.1	12.0	9.8	6.8
Limitations in PA	No	20706	68.3	63.6	66.6	72.3
	Yes	11299	31.7	36.4	33.4	27.7
Self-rated health	Not good	22455	68.6	74.8	71.4	63.0
	Good	9550	31.4	25.2	28.6	37.0

Note: PAG: physical activity guidelines - ≥ 150 minutes of moderate-intensity physical activity or ≥75 minutes of vigorous-intensity physical activity or an equivalent combination of moderate- and vigorous-intensity physical activity throughout the week.

The group of inactivity, insufficiency, and meeting physical activity guidelines: < 10, 10–149, ≥ 150 min/week of moderate-intensity equivalent physical activity in all domains. Col%: column %. All characteristics except for BMI were statistically significantly different among the three groups (*p* < 0.05).

### Total and domain-specific PA levels

The prevalence of individuals meeting PAG was 46.3% (Table 2). Women and the older age group had a lower prevalence (men and women aged 20–34 years: 63.5% and 53.2%; aged 65–79 years: 38.2% and 30.5%). The proportion of physically inactive individuals was high at 30.8%. Among all adults, 31.5% participated in at least 10 minutes of continuous LTPA per week, while 62.8% of men and 74.1% of women, and 77.4% of men and 88.1% of women aged 65–79, did not. Only 19% met the guidelines through LTPA, representing less than half of those who met the guidelines through PA in all domains. For men aged 65–79 and women aged 50–64, the corresponding prevalence was approximately 14%, and for women aged 65–79, it was 6.3%, equivalent to one-sixth of those meeting the PAG through all domains. Regarding TPA, 53.8% engaged in those activities and the prevalence of meeting the PAG through TPA was 29.1%. OPA participation was only reported by 8.7% and the prevalence of meeting the PAG through OPA was 5.7%. The proportion of meeting the PAG through LTPA and TPA, excluding OPA, was 43.3%.

**Table 2:** Total and domain-specific physical activity levels by gender and age group.

		Weighted %										
Physical activity domain	Level	Total	Men					Women				
		Age (year) 20–79	20–79	20–34	35–49	50–64	65–79	20–79	20–34	35–49	50–64	65–79
**Total**	Inactive (<10 min)	30.8	30.4	19.1	30.2	36.8	40.8	31.1	21.9	31.6	32.6	41.6
	Insufficient (10–149 min)	22.9	20.2	17.4	21.0	21.4	21.0	25.7	24.9	24.1	26.6	28.0
	Meeting PAG (≥150 min)	46.3	49.4	63.5	48.8	41.8	38.2	43.2	53.2	44.3	40.8	30.5
	150–299 min	19.5	18.0	20.2	18.4	16.0	16.6	21.1	25.1	20.8	20.4	16.9
	300–599 min	16.3	17.7	23.5	17.1	14.9	13.2	14.9	17.7	15.3	14.8	10.1
	600–899 min	5.5	6.6	8.8	6.2	5.8	5.0	4.3	6.0	4.7	3.6	2.2
	≥ 900 min	5.0	7.1	11.0	7.1	5.0	3.4	2.9	4.5	3.5	2.0	1.3
**Leisure/sports**	<10 min	68.5	62.8	53.1	61.4	67.0	77.4	74.1	67.2	70.0	76.5	88.1
	10–149 min	12.5	13.7	13.7	15.5	14.0	8.5	11.4	14.6	13.3	9.9	5.6
	≥150 min	19.0	23.5	33.2	23.1	19.0	14.0	14.5	18.2	16.7	13.5	6.3
	150–299 min	8.8	10.0	13.0	10.8	8.2	5.7	7.6	9.6	8.8	6.9	3.7
	300–599 min	6.9	8.6	12.5	8.3	6.8	4.9	5.1	6.5	5.8	5.0	2.2
	600–899 min	2.1	3.1	4.3	2.6	2.8	2.6	1.2	1.3	1.5	1.1	0.3
	≥ 900 min	1.2	1.8	3.3	1.4	1.1	0.8	0.5	0.9	0.6	0.4	0.1
**Transportation**	<10 min	46.2	50.2	37.4	55.5	55.7	52.3	42.1	33.9	46.0	42.2	46.9
	10–149 min	24.8	21.2	23.7	19.5	20.4	21.7	28.3	28.7	26.2	29.8	28.9
	≥150 min	29.1	28.6	38.8	25.0	23.9	26.0	29.6	37.4	27.8	28.0	24.2
	150–299 min	18.2	17.2	25.0	14.6	13.6	14.8	19.2	25.5	17.7	17.7	15.4
	300–599 min	8.5	8.8	11.2	7.9	7.6	8.5	8.2	8.9	8.1	8.1	7.1
	600–899 min	1.9	2.0	1.9	1.7	2.2	2.2	1.7	2.4	1.5	1.6	1.3
	≥ 900 min	0.6	0.7	0.8	0.7	0.5	0.5	0.5	0.5	0.5	0.5	0.4
**Work**	<10 min	91.3	88.9	85.4	85.6	92.8	95.9	93.7	90.1	92.0	96.7	96.9
	10–149 min	3.0	3.5	4.1	4.6	2.4	1.7	2.5	3.9	2.9	1.5	1.3
	≥150 min	5.7	7.6	10.5	9.8	4.8	2.4	3.8	6.0	5.2	1.8	1.8
	150–299 min	1.7	1.8	2.5	2.4	1.1	0.9	1.5	2.3	2.0	0.7	0.7
	300–599 min	1.6	2.1	3.1	2.9	1.1	0.5	1.0	1.6	1.4	0.4	0.5
	600–899 min	0.8	1.1	1.5	1.3	0.8	0.2	0.4	0.7	0.6	0.3	0.2
	≥ 900 min	1.7	2.5	3.4	3.2	1.8	0.7	0.9	1.4	1.1	0.4	0.5
**Leisure/sports and**	<10 min	33.1	33.6	22.0	35.1	39.2	42.2	32.6	24.0	33.6	33.4	42.4
**transportation**	10–149 min	23.6	20.8	18.5	21.8	21.6	21.0	26.4	26.1	25.0	27.1	28.3
	≥150 min	43.3	45.6	59.5	43.1	39.1	36.7	41.0	49.9	41.4	39.5	29.3
	150–299 min	19.9	18.5	21.5	18.6	16.3	16.7	21.4	25.9	21.1	20.5	16.7
	300–599 min	15.7	17.3	23.4	16.2	14.8	12.7	14.1	16.2	14.6	14.2	9.8
	600–899 min	4.8	5.8	8.3	4.9	5.0	4.7	3.7	5.5	3.6	3.4	2.1
	≥ 900 min	2.9	4.0	6.4	3.4	3.0	2.6	1.7	2.3	2.1	1.4	0.7

Note: All physical activity times in the table are the moderate-intensity equivalent minutes, summing the moderate-intensity activity time and the doubled vigorous-intensity activity time.

### Primary PA domain and contribution of each PA domain to total PA

Among those who were not inactive, the primary domain that occupied the majority of their PA time was transportation (59.0%) followed by leisure (31.4%), and work (7.9%) (Table 3). The proportion of individuals whose primary domain was transportation was higher among women and the elderly, reaching 82.1% among women aged 65–79. By contrast, the proportion of individuals whose primary domain was leisure was > 10%p lower in the age 65–79 age group compared with other age groups. Among those with a total PA time of <300 min/week, approximately 70% had TPA as their primary domain, while approximately 25% had LTPA. For those with 300–899 min/week, over 40% had either TPA or LTPA as their primary domain. For those with ≥900 min/week, 19% had TPA as their primary domain, while 40% had LTPA and OPA.

**Table 3:** Patterns of primary physical activity domain among those who are not inactive.

	Weighted %										
	Total	Men					Women				
	Age (year) 20–79	20–79	20–34	35–49	50–64	65–79	20–79	20–34	35–49	50–64	65–79
**Among those not inactive (≥10 min of physical activity)**											
Leisure/sports	31.4	37.7	38.9	39.8	38.0	27.5	24.9	26.2	29.4	24.3	13.5
Transportation	59.0	50.1	48.6	44.2	52.2	66.6	68.1	65.2	61.4	71.1	82.1
Work	7.9	10.4	10.7	14.2	7.8	4.6	5.3	6.9	6.8	3.1	3.7
Else	1.8	1.8	1.8	1.8	2.0	1.4	1.7	1.7	2.4	1.5	0.7
**Among those insufficient (10**–**149 min of physical activity)**											
Leisure/sports	24.0	32.3	26.9	39.7	34.8	18.2	17.5	20.2	23.7	14.5	8.9
Transportation	71.7	61.9	67.4	52.9	60.6	76.9	79.5	76.6	72.3	83.2	88.8
Work	3.5	5.1	5.1	6.9	3.9	3.8	2.2	2.6	3.0	1.3	1.8
Else	0.8	0.7	0.7	0.6	0.7	1.1	0.8	0.7	1.0	1.0	0.4
**Among those meeting the PAG (≥150 min of physical activity)**											
Leisure/sports	35.0	39.9	42.3	39.8	39.7	32.6	29.3	29.0	32.6	30.7	17.7
Transportation	52.7	45.3	43.4	40.5	47.9	60.9	61.3	59.9	55.5	63.2	75.9
Work	10.0	12.5	12.2	17.3	9.9	7.2	7.2	9.0	8.8	4.2	5.4
Else	2.2	2.3	2.1	2.4	2.6	2.2	2.2	2.1	3.1	1.9	0.9
**Among those with 150**–**299 min of physical activity time**											
Leisure/sports	25.8	30.6	27.6	34.7	32.8	22.5	21.7	19.9	26.0	22.9	13.3
Transportation	68.7	63.3	67.0	56.3	62.0	75.7	73.4	75.5	67.3	73.2	83.1
Work	3.7	4.2	3.4	7.2	2.7	1.5	3.3	3.8	3.9	2.5	2.9
Else	1.7	1.8	1.9	1.7	2.6	0.3	1.5	0.8	2.8	1.4	0.7
**Among those with 300**–**599 min of physical activity time**											
Leisure/sports	41.1	45.6	47.8	46.5	45.2	35.6	35.6	35.2	37.6	38.1	24.2
Transportation	49.1	43.9	41.6	39.3	47.1	58.3	55.4	52.7	52.2	56.3	69.6
Work	7.3	8.2	8.2	12.1	5.1	4.2	6.2	8.6	7.5	3.1	4.8
Else	2.5	2.3	2.3	2.1	2.6	1.9	2.8	3.5	2.7	2.5	1.3
**Among those with 600**–**899 min of physical activity time**											
Leisure/sports	45.9	48.9	54.7	44.5	45.2	50.5	41.2	45.0	43.2	39.4	22.8
Transportation	37.2	32.8	31.1	26.7	39.6	40.3	44.0	41.8	36.3	49.6	67.9
Work	13.3	14.9	11.8	22.5	13.6	6.8	10.8	8.7	16.1	7.6	7.7
Else	3.6	3.3	2.4	6.3	1.6	2.5	4.0	4.5	4.4	3.4	1.6
**Among those with ≥900 min of physical activity time**											
Leisure/sports	39.0	40.9	47.3	33.0	39.1	43.8	34.4	34.4	35.1	39.7	16.1
Transportation	18.9	15.0	13.8	13.9	14.7	29.4	28.5	24.9	25.4	35.1	44.6
Work	40.0	41.9	37.2	51.8	42.6	22.6	35.1	39.9	34.9	25.3	39.3
Else	2.1	2.1	1.7	1.4	3.6	4.1	2.0	0.8	4.7	.	.

Note: PAG: physical activity guidelines - ≥ 150 minutes of moderate-intensity physical activity or ≥75 minutes of vigorous-intensity physical activity or an equivalent combination of moderate- and vigorous-intensity physical activity throughout the week.

All physical activity times in the table are the moderate-intensity equivalent minutes, summing the moderate-intensity activity time and the doubled vigorous-intensity activity time.

The primary physical activity domain is the domain that accounts for the largest proportion of an individual’s total physical activity time. The ‘else’ category refers to the cases where ≥2 domains account for the largest proportion.

In terms of the contribution of each domain to the total PA time (Table 4), the proportion of individuals whose PA comprised more than half from each respective domain was 60.3% TPA, 32.4% LTPA, and 7.8% OPA. Additionally, the proportion of those whose PA comprised more than three-quarters from each domain was as follows: 51.4% TPA, 22.4% LTPA, and 5.4% OPA.

**Table 4:** Contribution of domain-specific physical activity to total physical activity among those who are not inactive.

		Weighted %										
		Total	Men					Women				
		Age (year) 20–79	20–79	20–34	35–49	50–64	65–79	20–79	20–34	35–49	50–64	65–79
**Leisure/sports**	0 percent	54.4	46.6	42.1	44.8	47.7	61.9	62.4	58.0	56.1	65.1	79.7
	1–24 percent	4.7	5.3	6.3	5.9	4.4	2.6	4.1	4.7	5.2	3.4	2.2
	25–49 percent	8.4	9.4	11.9	8.6	8.3	7.0	7.5	10.4	7.8	5.8	4.2
	50–74 percent	10.0	11.1	14.9	10.0	9.5	7.5	8.9	10.6	9.7	8.6	4.1
	75–100 percent	22.4	27.6	24.8	30.7	30.0	21.0	17.1	16.3	21.2	17.0	9.9
**Transportation**	0 percent	22.2	28.5	22.7	36.3	29.9	19.4	15.9	15.4	21.0	14.2	9.2
	1–24 percent	7.1	8.8	11.9	8.4	7.0	5.6	5.3	6.3	6.2	4.4	3.4
	25–49 percent	10.5	11.4	15.8	10.0	9.5	7.3	9.6	12.2	9.8	9.0	4.9
	50–74 percent	8.9	9.3	11.9	8.2	8.2	7.9	8.4	11.1	9.0	7.1	4.2
	75–100 percent	51.4	42.0	37.8	37.2	45.5	59.8	60.8	55.0	53.9	65.2	78.4
**Work**	0 percent	87.5	84.1	82.0	79.4	88.6	93.1	90.9	87.4	88.2	95.0	94.7
	1–24 percent	2.0	2.6	3.6	2.8	1.8	1.1	1.5	2.3	1.9	0.6	0.6
	25–49 percent	2.7	3.0	3.8	3.8	1.8	1.2	2.3	3.3	3.0	1.3	0.9
	50–74 percent	2.4	3.0	3.7	3.5	2.2	1.2	1.9	2.6	2.2	1.2	1.4
	75–100 percent	5.4	7.3	6.9	10.6	5.6	3.5	3.4	4.4	4.6	1.9	2.4

### Associations between PA patterns and good SRH

Table 5 shows the association between total PA time or domain-specific PA time and SRH. After adjusting for various sociodemographic factors, the longer total aerobic PA time was associated with the higher ORs for having good SRH (*p* for trend <0.0001; ORs from 1.31 for 150–299 to 2.02 for ≥900 min/week, compared to the inactive). In a model incorporating three PA domains together, the longer LTPA time was associated with higher ORs for good SRH. Compared with the individuals who had no LTPA, those with leisure time ranging from 10–149 min/week up to ≥900 min/week exhibited ORs increasing from 1.41 to 3.90. TPA time showed a weak association (all ORs for groups by PA time were <1.23), while OPA time showed no association.

**Table 5:** Association between self-rated health and domain-specific physical activity level, pattern, and contribution to total physical activity.

	Level	N	Good self-rated health (%)	Gender and age-adjusted OR (95% CI)	Multivariate OR (95% CI)
**Total physical activity**	Inactive (<10 min)	10532	25.2	1 (ref)	1 (ref)^a^
	Insufficient (10–149 min)	7512	28.6	1.17 (1.08-1.27)	1.11 (1.02-1.21)
	150-299 min	6135	33.0	1.39 (1.28-1.50)	1.31 (1.20-1.42)
	300-599 min	4871	37.7	1.65 (1.51-1.80)	1.52 (1.39-1.67)
	600-899 min	1584	41.7	1.89 (1.66-2.14)	1.75 (1.53-1.99)
	≥900 min	1371	45.1	2.07 (1.80-2.39)	2.02 (1.75-2.33)
**Domain-specific physical activity**					
**Leisure/sports**	<10 min	22824	26.4	1 (ref)	1 (ref)^b, c^
	10-149 min	3763	37.1	1.55 (1.43-1.69)	1.41 (1.29-1.54)
	150-299 min	2555	42.0	1.86 (1.68-2.05)	1.66 (1.49-1.84)
	300-599 min	1951	45.7	2.12 (1.90-2.38)	1.90 (1.68-2.14)
	600-899 min	607	52.9	2.80 (2.32-3.37)	2.48 (2.04-3.02)
	≥900 min	305	63.2	4.06 (3.05-5.41)	3.90 (2.90-5.25)
**Transportation**	<10 min	15033	29.5	1 (ref)	1 (ref)^b, c^
	10-149 min	8035	31.0	1.08 (1.00-1.16)	1.04 (0.96-1.12)
	150-299 min	5576	34.8	1.21 (1.12-1.31)	1.13 (1.04-1.23)
	300-599 min	2612	34.7	1.22 (1.10-1.35)	1.12 (1.00-1.25)
	600-899 min	581	36.2	1.29 (1.06-1.58)	1.23 (0.99-1.52)
	≥900 min	168	33.6	1.14 (0.77-1.68)	1.09 (0.73-1.62)
**Work**	<10 min	29553	31.5	1 (ref)	1 (ref)^b, c^
	10-149 min	876	26.8	0.71 (0.59-0.86)	0.71 (0.58-0.86)
	150-299 min	475	35.6	1.08 (0.86-1.35)	1.01 (0.80-1.28)
	300-599 min	433	32.6	0.89 (0.70-1.14)	0.90 (0.70-1.15)
	600-899 min	207	33.9	0.95 (0.68-1.32)	0.97 (0.67-1.40)
	≥900 min	461	31.0	0.83 (0.66-1.05)	0.85 (0.67-1.09)
**Primary domain of physical activity**					
	Inactive	10532	25.2	1 (ref)	1 (ref)^d^
	Leisure/sports	6315	43.8	2.08 (1.92-2.26)	1.48 (1.33-1.64)
	Transportation	13261	29.8	1.22 (1.14-1.30)	1.01 (0.93-1.10)
	Work-related	1535	28.7	1.04 (0.90-1.20)	0.76 (0.64-0.90)
	Else	362	38.0	1.67 (1.30-2.16)	1.15 (0.88-1.51)
**Contribution of domain-specific activity**					
**Leisure/sports**	Inactive	10532	25.2	1 (ref)	1 (ref)^d, e^
	0 percent	12292	27.4	1.09 (1.01-1.17)	0.96 (0.88-1.05)
	1-24 percent	948	36.0	1.49 (1.27-1.74)	1.07 (0.89-1.28)
	25-49 percent	1691	41.2	1.85 (1.63-2.10)	1.35 (1.16-1.56)
	50-74 percent	1990	44.0	2.06 (1.83-2.33)	1.44 (1.24-1.67)
	75-100 percent	4552	43.5	2.10 (1.92-2.30)	1.60 (1.44-1.79)
**Transportation**	Inactive	10532	25.2	1 (ref)	1 (ref)^d, e^
	0 percent	4501	38.2	1.68 (1.54-1.84)	1.35 (1.22-1.50)
	1-24 percent	1382	46.0	2.22 (1.93-2.55)	1.57 (1.32-1.86)
	25-49 percent	2078	42.4	1.93 (1.72-2.18)	1.40 (1.21-1.62)
	50-74 percent	1827	39.6	1.75 (1.55-1.98)	1.30 (1.12-1.50)
	75-100 percent	11685	28.3	1.15 (1.07-1.23)	1.01 (0.92-1.10)
**Work**	Inactive	10532	25.2	1 (ref)	1 (ref)^d, e^
	0 percent	19021	34.7	1.49 (1.40-1.59)	1.14 (1.05-1.24)
	1-24 percent	396	35.6	1.35 (1.06-1.74)	0.84 (0.64-1.11)
	25-49 percent	519	36.1	1.42 (1.15-1.76)	0.93 (0.74-1.17)
	50-74 percent	463	32.5	1.22 (0.97-1.53)	0.78 (0.60-1.01)
	75-100 percent	1074	26.1	0.90 (0.75-1.07)	0.61 (0.50-0.75)

Note: All multivariate ORs were adjusted for gender, 10-year age group, educational level, household income quartile, occupation, residential area, BMI group, the number of chronic diseases, and limitations in PA.

OR (95% CI) for trend: ^a^1.15 (1.13–1.18, *p* < 0.0001); ^c^1.27 (1.23–1.30, *p* < 0.0001) for leisure/sports, 1.05 (1.02–1.08, *p* = 0.0021) for transportation, 0.97 (0.93–1.00, *p* = 0.0698) for work; ^e^1.10 (1.08–1.12, *p* < 0.0001) for leisure/sports, 0.98 (0.97–1.00, *p* = 0.0135) for transportation, 0.95 (0.92–0.97, *p* < 0.0001) for work.

^b^Multivariate ORs of the domain-specific physical activity were additionally adjusted for other domain-physical activities (i.e., mutually adjusted in a model)

^d^Multivariate ORs of the primary PA domain and the contribution of each domain-specific PA to an individual’s total PA time were additionally adjusted for total physical activity level

The similar results were shown for the primary PA domain. The odds for good SRH were 1.48 times higher among those whose primary domain was leisure/sports (95% CI 1.33–1.64) compared to inactive individuals, but 0.76 times lower among those whose main domain was work (95% CI 0.64–0.90). Transportation showed no significant association. Regarding the contribution of domain-specific time to the total PA, a higher contribution of LTPA was associated with good SRH. Individuals with over 75% of their total PA time spent on LTPA had odds of good SRH up to 1.60 times greater than inactive individuals. However, for TPA, the OR was highest when it constituted 1–24% of the total PA time (OR 1.57), and the higher the composition, the lower the ORs. For work activities, the OR of good SRH was highest at 1.14 in individuals who had no OPA, while those who spent ≥75% of the total PA time in OPA showed an inverse association, reducing their OR of good SRH (OR 0.61, 95% CI 0.50–0.75).

## Discussion

This study examined the levels and patterns of domain-specific PA among South Korean adults and evaluated the relationship between PA domains and SRH as a surrogate for health. We found that the globally low levels of PA among Korean adults, as reported by the WHO, are primarily attributed to low levels of work-related PA. The primary PA domain for Korean adults was transportation, which was particularly pronounced among women and the elderly. The prevalence of meeting PAG solely through LTPA was less than the half level of meeting PAG through PA in all domains. Regarding good SRH, only LTPA demonstrated clear and dose-response relationships.

The proportion of adults aged 20–79 in Korea who met the PAG from 2016 to 2021 was 46.3%, which aligns with previous WHO reports [3]. For each year from 2019 to 2021, South Korea consistently ranked sixth out of 195 countries for the prevalence of insufficient PA (not meeting the PAG) among adults— > 10%p higher than that of Japan and Brazil, > 20%p higher than that of the United States (U.S.) and Vietnam, and >30%p higher than Mongolia, Singapore, China, and the United Kingdom.

Over 90% of individuals did not undertake in OPA. The proportion of those who participated in OPA for ≥10 min/week or met the PAG solely through OPA were 8.7% and 5.7%, respectively. These figures are significantly lower compared with countries such as the U.S. (over 35% met the PAG through OPA), China (66.8% OPA for ≥10 min/week), and Japan (16.5% OPA for ≥10 min/week among those aged 30–59) [15–22]. The proportion of individuals in Korea for whom OPA is the primary domain was around 10%, even among men aged<50. In China and Brazil, the mean contribution of OPA was 55% and 70%, respectively [18,20]. Excluding OPA, the proportion of meeting the PAG through LTPA and TPA only decreased by approximately 5%. Therefore, one likely reason for the low proportion of adults meeting the PAG in Korea is the low level of OPA. Such very low levels of OPA may have reflected the combined influence of Korea’s labor and residential environments, as well as the characteristics of the measurement tool. Korea has an employment structure largely centered on clerical, professional, and service occupations [23]. In addition, within manual occupations, the proportion of plant and machine operators and assemblers is not negligible, and delivery workers have increased within elementary occupations [24,25]. These jobs may involve long working hours, but they do not necessarily entail sustained moderate- to vigorous-intensity PA. In addition, because the GPAQ includes household chores within the occupational domain, Korea’s apartment- and multi-family housing–oriented residential structure, accounting for approximately 63% of households and 78% of the total housing stock, may have reduced opportunities for relatively physically demanding domestic activities that are more common in detached-house settings, such as yard work, snow removal, and exterior home maintenance [26]. Moreover, because the GPAQ captures only PA accumulated in bouts of at least 10 consecutive minutes, intermittent or time-fragmented work and household activities that are common in Korea, such as on-demand delivery work, may have involved frequent movement without meeting the questionnaire’s duration and intensity thresholds, and therefore may not have been captured as OPA.

However, the significance of this issue may depend on OPA’s health effects. In this study, OPA showed no significant association with good SRH for domain-specific PA time and a negative association regarding the primary domain or the contribution to overall PA. These findings are consistent with results from other Korean studies but do not align with the current general understanding of the health effects of OPA. This discrepancy may partly reflect the nature of the outcome measure. SRH is not merely a report of physical health status but rather a summary indicator that incorporates subjective, social, and psychosocial experiences and cognitive appraisal [27]. In this context, negative experiences associated with OPA, such as long working hours, repetitive tasks, high physical demands, insufficient recovery, fatigue, and psychological strain, may have been more readily reflected in SRH than in objective health indicators. At the same time, because SRH is also a well-established predictor of morbidity and mortality, this finding should not be dismissed as merely perceptual. Nevertheless, the overall health effects of OPA remain controversial. The 2020 WHO Physical Activity and Sedentary Behavior Guideline Development Group (GDG) concluded that PA in different domains, including high levels of OPA, can be beneficial for health and stated that it is not currently possible to differentiate the effect of PA in different domains on various health outcomes [1]. However, they noted that higher levels of OPA may be associated with an increased risk of osteoarthritis, poor sleep quality, and all-cause mortality in males [1,4]. Some researchers have recently presented such evidence, suggesting that OPA exhibits different health effects compared to LTPA, referring to it as the PA paradox [4–6,28–30]. They attributed this to the characteristics of OPA, such as working in monotonous or awkward postures, prolonged duration, and insufficient recovery time [30]. One study argued that this issue might stem from methodological problems in research but noted that the decreasing number of heavy workers could reduce the practical relevance of OPA to health. They also mentioned that workers in all sectors might need to rely on leisure time to achieve the PA necessary for maintaining health in the future [31]. The GDG also noted that in high-income countries, the health effects of OPA are inconclusive, and may be confounded by socioeconomic position or exposure to hazardous working conditions [32]. Therefore, the health effects of OPA may vary depending on a country’s industrial configuration and patterns of OPA demands. The potential loss of health benefits resulting from the high prevalence of insufficient PA in Korea, which was likely attributable to low OPA levels, remains unclear. However, when considering the relationship between OPA and good SRH reported in this study, these losses are considered likely to be minimal.

Unlike work-related activity, TPA was the primary PA domain among Korean adults in our study, accounting for half of the men and two-thirds of the women. This emphasis on TPA as the primary PA domain was particularly pronounced among women, the elderly, and those with insufficient PA. While 29% met the PAG through TPA alone—higher than the 10–15% reported in the U.S. [15–17] —54% undertook at least 10 minutes of TPA per week, a rate similar to those in China (52%) and Japan (45% among those aged 30–59). A plausible explanation for the predominance of TPA is the accumulation of utility walking in a highly urbanized and transit-oriented environment. In Korea, more than three-quarters of the population live in urban centres [33]. In metropolitan areas such as Seoul, dense residential and commercial destinations and integrated public transport networks may facilitate TPA [34]. Such environments may promote TPA not only through commuting to work or school, but also through access, egress, and transfers associated with public transport use in routine trips. Rural Korean villages, too, have traditionally shown a clustered settlement pattern, which may allow walking to nearby community facilities and routine destinations [35]. The larger role of TPA among women and older adults in our study is also consistent with travel patterns in Korea, where women make a higher share of non-work trips, rely more on public transport for everyday travel, and have a lower car commuting share than men, whereas older adults have a higher walking mode share [33,34]. Overall, TPA in Korea may reflect the structure of everyday mobility rather than intentional engagement in health-enhancing PA.

Active commuting and transport by walking or cycling are considered effective strategies for increasing PA levels for health benefits [7–9]. TPA, particularly walking, is estimated to be 3.5 METs and 4.3 METs in adults aged 15–59 and ≥60, respectively, falling within the low end of the MET range (3.0–6.0) for moderate-intensity PA [1,36,37]. Therefore, activities like walking for transportation may have different health effects compared with higher-intensity LTPA or exercise. In a systematic review, TPA and moderate-intensity activity were found to have a smaller influence on reducing all-cause mortality compared with LTPA and vigorous-intensity activity [38]. Additionally, a study, an evidence for the WHO 2020 guideline showed that active commuting reduces the risk of all-cause mortality by 8%. However, cycling significantly reduced overall mortality, while working did not, which was attributed to its higher energy expenditure intensity and greater potential for improving fitness [7]. Another study found that the health benefits of TPA could vary depending on regional characteristics, including the amount of PA, road traffic fatalities, and air pollution exposure [8]. Notably, the question about TPA in the GPAQ differs from those about moderate-intensity activity in the other two domains in that it does not include descriptions or examples such as “increasing breathing” or “slightly increasing breathing or heart rate like brisk walking.” In these aspects, TPA may have lower intensity than moderate-intensity activity in other domains, potentially reflecting regional characteristics that could result in varying health benefits.

Although 46.3% meet the PAG through PA across all domains, the proportion meeting PAG through LTPA alone was only 19%, which is lower than in the U.S. (approximately 35%) [15–17]. The proportion participating in LTPA for ≥10 min/week was 31.5%, which was higher than in Japan (26% for ages 30–59) and China (17%) [18,19]. This pattern suggests that, in Korea, small amounts of LTPA may be maintained, but do not amount to sufficient levels of sustained and structured LTPA. Korea has long been noted for long working hours among OECD countries [39], and the National Sports Participation Survey has also consistently reported lack of time as the most common reason for non-participation in regular sports and exercise [40]. Under these conditions, activities that can be incorporated into daily routines may be more feasible than LTPA, which requires dedicated time and planning. Meanwhile, the same survey also identified lack of interest in sports and exercise as another major reason [40], which may suggest that, in some groups, structured leisure exercise or organized sports activities have not become familiar and accessible forms of everyday life. This may also be partly related to the very high prevalence of insufficient PA among Korean adolescents [41]. Among older adults, health problems were reported as a major reason [40], and barriers to exercise have also been reported to include limited information and knowledge about exercise, inadequate places and facilities, and concerns about injury or pain [42]. These factors may have made organized, age- and function-appropriate LTPA more difficult [42–44]. In addition, because LTPA may depend more heavily than TPA on time, facilities, financial cost, and social and environmental resources, this background may partly explain why the actual PA pattern of Korean adults is relatively more weighted toward TPA than toward structured exercise.

Regarding good SRH, LTPA showed a clear dose-response relationship in all aspects (PA time, contribution to total PA, and primary domain). Another Korean study also showed a similar association with SRH [45]. LTPA is the PA domain that is most consistently and strongly associated with mortality and health [38] and has shown beneficial effects for both physical and mental health [16,46,47]. Considering the psychosocial mechanisms of PA such as enhancing self-efficacy, confidence-building opportunities, social interactions, and distraction from stress, and the differences in motivation of participation, the differences in health effects observed across domains seem logical. The low rate of meeting PAG through LTPA in Korea is regrettable, despite its unique health benefits and inclusive range including relatively easy recreational activities, such as brisk walking, that are suitable even for older adults. It is plausible that the high levels of TPA among Korean adults may inadvertently contribute to a lack of focus on the promoting LTPA.

In the WHO’s STEPwise approach to NCD risk factor surveillance, PA is measured using the GPAQ to determine total PA time from all domains. However, in the U.S., the Healthy People 2030 objectives measure PA using LTPA time from the National Health Interview Survey [48]. While this may be seen as simply a matter of respondent burden or survey space, it affects estimates of meeting PA guidelines and highlights that total PA and domain-specific PA patterns can differ [49]. Given that health effects may vary by PA domain, it is important to monitor both total PA and domain-specific PA at a national level, which is also useful for monitoring differences in PA levels and domain patterns according to socioeconomic status.

This study has several limitations. First, unlike previous guidelines, the WHO 2020 Physical Activity Guidelines [1,2] do not require PA to be undertaken in bouts of at least 10 minutes. However, the GPAQ only mentions PA that lasts at least 10 consecutive minutes, meaning that the amount of domain-specific PA measured in this study using the GPAQ and the contribution of domain-specific PA based on this measure may not accurately reflect overall PA, particularly for TPA or specific OPA. Additionally, the primary PA domain and the contribution of each domain were only analyzed among those who were not inactive, i.e., those who engaged in at least 10 minutes of continuous activity per week, in any domain. Additionally, this study has a cross-sectional design, which does not permit for the determination of temporal or causal relationships between domain-specific PA and health. Furthermore, we used SRH as a surrogate for overall health, instead of using all-cause mortality or disease incidence. Nevertheless, SRH is a strong indicator of mortality and morbidity in both adults and the elderly and is widely used as a simple proxy for overall health [50,51].

## Conclusions

This study examined the patterns of PA domains, with results suggesting that the low levels of PA among Korean adults were primarily due to low levels of OPA. The primary PA domain was transportation. Even among those individuals who met the PAG, a significant proportion did not meet them through LTPA. Regarding SRH used as a proxy for health, only LTPA showed a clear dose-response relationship in all aspects. In summary, in order to promote PA for health benefits, it is necessary to monitor not only total activity levels but also activity patterns in different domains due to potential differences in health benefit and Korean societal patterns.
