# Supplementary material for: Do South Korean adults have insufficient levels of physical activity? Domain patterns and associations with self-rated health
Source: PLoS One. 2026 Jun 22;21(6):e0352382. doi: 10.1371/journal.pone.0352382 (PMC13286163; doi:10.1371/journal.pone.0352382)
Supplement: Attachment — Submitted filename: PAA_Response_to_Reviewers.docx [file pone.0352382.s001.docx]

**Response to Reviewers**

Thank you for giving us the opportunity to submit a revised draft of the manuscript “Do South Korean adults have insufficient levels of physical activity? Domain patterns and associations with self-rated health” (PONE-D-24-49285) for publication in PLOS ONE. Please see below for a point-by-point response to the reviewers’ comments and concerns.

**Reviewer #1: This study examined the physical activity levels of adults in South Korea and whether domain-specific physical activity is associated with self-rated health. The study has scientific relevance. Below are my comments.**

**1. In my opinion, the author should clearly indicate that the study was among adults in South Korea – in the title and throughout the article.**

**Author response:** Thank you for this helpful comment. In response, we revised the title, Abstract, Methods, and Discussion to clarify that this study was conducted among South Korean adults.

**Title:**
(Original) Do Koreans have insufficient levels of physical activity? Domain patterns and their association with self-rated health
(Revised) Do South Korean adults have insufficient levels of physical activity? Domain patterns and associations with self-rated health

**Abstract (Methods):**
(Original) “Data from 32,005 adults aged 20–79 from the Korean National Health and Nutrition Examination Survey (2016–2021) were analyzed.”
(Revised) “Data from 32,005 South Korean adults aged 20–79 from the Korean National Health and Nutrition Examination Survey (2016–2021) were analyzed.”

The final sentence of the **Introduction** already specified that the study population was South Korean adults:
“Therefore, this study aimed to evaluate domain-specific PA patterns, including PA levels and the prevalence of meeting PAG based on these activities, among South Korean adults to explain the high prevalence of insufficient PA, and to assess their association with good self-rated health (SRH) as a proxy for overall health outcomes.”

In the **Data source and study subjects** subsection of the **Methods**, we revised the following sentence:
(Original) “Of the 35,688 individuals aged 20–79 who participated in the health surveys during 2016–2021, 3,388 subjects ... were excluded.”
(Revised) “Of the 35,688 South Korean adults aged 20–79 who participated in the health surveys during 2016–2021, 3,388 subjects ... were excluded.”

In the first sentence of the **Discussion**, we revised:
(Original) “This study examined the levels and patterns of domain-specific PA among Korean adults and evaluated the relationship ... ”
(Revised) “This study examined the levels and patterns of domain-specific PA among South Korean adults and evaluated the relationship ...”

**2. Statistical Analysis: The author should provide information on whether the data met the assumptions of the multivariate analysis. The results of diagnostics tests for this purpose should be added to the analysis section.**

**Author response:** Thank you for this important comment. Our main multivariable analysis used the SAS SURVEYLOGISTIC procedure with good self-rated health as a binary outcome. Because all explanatory variables and covariates were modeled as categorical variables, assumptions related to linearity in the logit for continuous predictors were not applicable. In addition, all analyses accounted for the complex sampling design of the KNHANES. To clarify this, we added a corresponding statement to the Statistical Analysis section.

Because the SURVEYLOGISTIC procedure does not directly provide variance inflation factor (VIF)-based diagnostics for multicollinearity, we conducted an additional check using models with the same predictor structure outside the complex survey framework. This supplementary assessment did not indicate problematic multicollinearity among predictors (all VIFs < 5). We therefore considered multicollinearity unlikely to have materially affected the main findings.

The following sentence was added to the Statistical Analysis section:

“As good SRH was analyzed as a binary outcome and all explanatory variables were entered as categorical variables, logistic regression was considered appropriate for the analyses, and assumptions related to linearity in the logit for continuous predictors were not applicable.”

**3. In my opinion, the Discussion should offer more insight into the explanations and implications of the findings. This should be done in the context of past research with appropriate citations.**

**Author response:** We thank the reviewer for these insightful comments, which helped us to further develop and expand the Discussion, particularly regarding the prevalence and patterns of domain-specific physical activity in the Korean context. We have revised the Discussion accordingly, as detailed below. **We respond to each point as follows:**

**a. Why is it that South Korea ranks high for insufficient physical activity among adults compared to other countries?**

**Author response:** Unlike comments b–e, we believe that this point was partly addressed in the original Discussion, where we stated: “Excluding OPA, the proportion of meeting the PAG through LTPA and TPA only decreased by approximately 5%. Therefore, one likely reason for the low proportion of adults meeting the PAG in Korea is the low level of OPA.” The reasons underlying the low level of OPA were specifically addressed in response to comment b, where we have provided additional explanation.

**b. Why is it that over 90% of Koreans do not undertake OPA?**

**Author response:** In response to this comment, we added the following explanation to the Discussion:

“Such very low levels of OPA may have reflected the combined influence of Korea’s labor and residential environments, as well as the characteristics of the measurement tool. Korea has an employment structure largely centered on clerical, professional, and service occupations [23]. In addition, within manual occupations, the proportion of plant and machine operators and assemblers is not negligible, and delivery workers have increased within elementary occupations [24, 25]. These jobs may involve long working hours, but they do not necessarily entail sustained moderate- to vigorous-intensity PA. In addition, because the GPAQ includes household chores within the occupational domain, Korea’s apartment- and multi-family housing–oriented residential structure, accounting for approximately 63% of households and 78% of the total housing stock, may have reduced opportunities for relatively physically demanding domestic activities that are more common in detached-house settings, such as yard work, snow removal, and exterior home maintenance [26]. Moreover, because the GPAQ captures only PA accumulated in bouts of at least 10 consecutive minutes, intermittent or time-fragmented work and household activities that are common in Korea, such as on-demand delivery work, may have involved frequent movement without meeting the questionnaire’s duration and intensity thresholds, and therefore may not have been captured as OPA.”

**c. Are there any Korea-specific factors that explain the associations of OPA with self-rated health?**

**Author response:** We thank the reviewer for this thoughtful comment. In the revised Discussion, we added a context-specific interpretation of the inverse or null association between OPA and good SRH, emphasizing that SRH reflects not only physical health status but also subjective, social, and psychosocial experiences, as well as cognitive appraisal. Thus, adverse experiences associated with OPA may have been more readily reflected in SRH than in more objective health outcomes. We also considered whether culturally specific perceptions of occupation or manual work in Korea might have partly influenced SRH reporting, but we found it difficult to support this interpretation with direct evidence. Therefore, we did not emphasize this point in the revised manuscript. The revised text is as follows:

“This discrepancy may partly reflect the nature of the outcome measure. SRH is not merely a report of physical health status but rather a summary indicator that incorporates subjective, social, and psychosocial experiences and cognitive appraisal [27]. In this context, negative experiences associated with OPA, such as long working hours, repetitive tasks, high physical demands, insufficient recovery, fatigue, and psychological strain, may have been more readily reflected in SRH than in objective health indicators. At the same time, because SRH is also a well-established predictor of morbidity and mortality, this finding should not be dismissed as merely perceptual. Nevertheless, the overall health effects of OPA remain controversial.”

**d. Why do South Koreans accumulate physical activity primarily through transportation? And why is this the case mostly among women, the elderly, and those with insufficient physical activity?**

**Author response:** We appreciate the reviewer’s comment, which prompted us to consider and describe the Korean contextual factors relevant to this finding. In response, we added the following discussion:

“A plausible explanation for the predominance of TPA is the accumulation of utility walking in a highly urbanized and transit-oriented environment. In Korea, more than three-quarters of the population live in urban centres [33]. In metropolitan areas such as Seoul, dense residential and commercial destinations and integrated public transport networks may facilitate TPA [34]. Such environments may promote TPA not only through commuting to work or school, but also through access, egress, and transfers associated with public transport use in routine trips. Rural Korean villages, too, have traditionally shown a clustered settlement pattern, which may allow walking to nearby community facilities and routine destinations [35]. The larger role of TPA among women and older adults in our study is also consistent with travel patterns in Korea, where women make a higher share of non-work trips, rely more on public transport for everyday travel, and have a lower car commuting share than men, whereas older adults have a higher walking mode share [33, 34]. Overall, TPA in Korea may reflect the structure of everyday mobility rather than intentional engagement in health-enhancing PA.”

**e. Why is it that only a small proportion of South Koreans meet the physical activity guidelines with LTPA?**

**Author response:** We thank the reviewer for this important comment. In response, we added a more focused discussion of why only a small proportion of Korean adults meet the physical activity guidelines through LTPA, drawing primarily on findings from the Korean Ministry of Culture, Sports and Tourism’s Sports Participation Survey, which we considered relevant to the interpretation of low LTPA in this study. The revised text is as follows:

“This pattern suggests that, in Korea, small amounts of LTPA may be maintained, but do not amount to sufficient levels of sustained and structured LTPA. Korea has long been noted for long working hours among OECD countries [39], and the National Sports Participation Survey has also consistently reported lack of time as the most common reason for non-participation in regular sports and exercise [40]. Under these conditions, activities that can be incorporated into daily routines may be more feasible than LTPA, which requires dedicated time and planning. Meanwhile, the same survey also identified lack of interest in sports and exercise as another major reason [40], which may suggest that, in some groups, structured leisure exercise or organized sports activities have not become familiar and accessible forms of everyday life. This may also be partly related to the very high prevalence of insufficient PA among Korean adolescents [41]. Among older adults, health problems were reported as a major reason [40], and barriers to exercise have also been reported to include limited information and knowledge about exercise, inadequate places and facilities, and concerns about injury or pain [42]. These factors may have made organized, age- and function-appropriate LTPA more difficult [42–44]. In addition, because LTPA may depend more heavily than TPA on time, facilities, financial cost, and social and environmental resources, this background may partly explain why the actual PA pattern of Korean adults is relatively more weighted toward TPA than toward structured exercise.”

We thank the reviewer again for these valuable and constructive comments, which helped us improve and strengthen the manuscript.

**Journal Requirement #2:**

We thank the editor for this instruction. In response, we have deposited the minimal analytic dataset and all relevant code necessary to reproduce the study findings in a public repository (figshare) and have obtained a DOI (https://doi.org/10.6084/m9.figshare.32098204).

We have updated the Data Availability Statement accordingly.
