# Supplementary material for: Do South Korean adults have insufficient levels of physical activity? Domain patterns and associations with self-rated health
Source: PLoS One. 2026 Jun 22;21(6):e0352382. doi: 10.1371/journal.pone.0352382 (PMC13286163; doi:10.1371/journal.pone.0352382)
Supplement: Attachment — Submitted filename: Review_Kong2026.docx [file pone.0352382.s002.docx]

Title : **Do South Korean adults have insufficient levels of physical activity ? Domain patterns and associations with self-rated health**

Kong et al. present a manuscript examining domain-specific physical activity (PA) patterns among South Korean adults and their associations with self-rated health (SRH). Given the high prevalence of insufficient PA and the limited monitoring of domain-specific PA patterns in South Korea, the topic is relevant and timely. The study aimed to evaluate domain-specific PA patterns and investigate their associations with SRH as a proxy for overall health outcomes.

Overall, the manuscript addresses an important public health issue, and the analyses appear to adequately respond to the study objectives. The manuscript is generally well organized, and the methodology is appropriate. However, several sections would benefit from additional clarifications, improved wording, and structural adjustments to enhance readability and scientific precision. Please find below several comments and suggestions that may help strengthen the manuscript.

## General Comments

1. It would be recommended to consistently use the past tense throughout the manuscript to ensure coherence with the rest of the text, especially in the methods and results sections.
2. Please ensure that every instance of “p” referring to p-values is italicized throughout the manuscript.

## Background

3. Lines 40–47: It would be helpful to provide brief definitions and/or examples of the different PA domains (e.g., LTPA, OPA, TPA) for readers who may be less familiar with these concepts.
4. Line 48: The statement indicating that “South Korea has an exceptionally high prevalence of insufficient PA among adults” would benefit from supporting statistics and appropriate references.
5. Lines 49–50: It is unclear what criteria South Korea is being ranked on. Additional clarification would improve readability and comprehension.

## Methods

6. Line 63: Please define “KNHANES” at first mention and provide the appropriate reference.
7. Line 64: Since the study period is reported as 2016–2021, it is unclear whether KNHANES is still ongoing. If the data collection period is completed, “aims” should likely be replaced with “aimed.”
8. Lines 63–70: This paragraph would benefit from further clarification. It is currently difficult to determine whether KNHANES refers to a study, a survey, or a database.
9. Line 75: Please use the abbreviation “PA” consistently throughout the manuscript.
10. Line 90: There appears to be an extra character/symbol in the sentence regarding total PA time estimation.
11. Lines 92–94: If this paragraph refers to GPAQ scoring, it may be preferable to avoid a line break. Otherwise, additional clarification would be appreciated.
12. Lines 101–104: Please specify which health-related factors are assessed by the SRH scale.
13. Study variables: It may improve readability to introduce subheadings for each evaluated variable or assessment tool. Additionally, it would be useful to specify whether the measures were self-reported, interview-based, or assessed using another format.
14. Lines 109–112: No chronic mental health conditions appear to be included among the chronic diseases assessed. While this may reflect limitations of the existing database, acknowledging this point in the manuscript would strengthen the discussion, especially given the references to mental health in the introduction.
15. Lines 121–127: This paragraph could benefit from clearer wording and restructuring to facilitate reader comprehension.

## Results

16. Tables: Please indicate the units associated with the reported values directly in the column headings (e.g., %, n).
17. Tables: It would also be helpful to include the mention “Note:” directly below the tables in the legends.
18. To improve readability, it is recommended to organize the Results section into clear subsections. At minimum, separate subsections could be created for participant characteristics and for each study objective. For example :
    1.1 ***Sociodemographic and health-related characteristics of participants***
    1.2 ***Evaluation of domain-specific PA patterns***
        1.2.1 *PA levels*
        1.2.2 *Prevalence of meeting PAG*
    1.3 ***Assessment of domain-specific PA patterns association with SRH for overall outcomes***
        1.3.1 *Total PA x SRH*
        1.3.2 *Domain-specific x SRH*

## Discussion

19. Lines 379–385: Additional references regarding the benefits of PA on mental health would strengthen this section.
20. Regarding the limitations associated with the GPAQ assessment tool, the authors may wish to consider discussing the SIMPAQ questionnaire as a potential avenue for future research. The SIMPAQ has demonstrated international validity and reliability in both general and mental health populations and may provide a more precise assessment of PA and sedentary behaviors. Suggested reference: Rosenbaum, S., Morell, R., Abdel-Baki, A., Ahmadpanah, M., Anilkumar, T. V., Baie, L., et al. (2020). Assessing physical activity in people with mental illness: 23-country reliability and validity of the Simple Physical Activity Questionnaire (SIMPAQ). BMC Psychiatry, 20(1), 108. <https://doi.org/10.1186/s12888-020-2473-0>

Thank you for the opportunity to review this interesting manuscript. I hope these comments and suggestions will be helpful to the authors in strengthening the clarity and overall quality of the paper.
