# Supplementary material for: Do South Korean adults have insufficient levels of physical activity? Domain patterns and associations with self-rated health
Source: PLoS One. 2026 Jun 22;21(6):e0352382. doi: 10.1371/journal.pone.0352382 (PMC13286163; doi:10.1371/journal.pone.0352382)
Supplement: Attachment — Submitted filename: PAA_Response_to_Reviewers_R2.docx [file pone.0352382.s003.docx]

**Response to Reviewers**

Thank you for giving us the opportunity to submit a revised version of the manuscript, “Do South Korean adults have insufficient levels of physical activity? Domain patterns and associations with self-rated health” (PONE-D-24-49285R1), for publication in PLOS ONE. We sincerely thank the reviewer for the careful evaluation of our manuscript and for the constructive comments and suggestions. Please see below for a point-by-point response to the reviewer’s comments.

## General Comments

1. It would be recommended to consistently use the past tense throughout the manuscript to ensure coherence with the rest of the text, especially in the methods and results sections.

**Author response:** Thank you for this comment. We reviewed the manuscript to improve tense consistency, particularly in the Methods and Results sections. We retained the present tense for general descriptions that remain currently valid, such as descriptions of the ongoing KNHANES and its standard procedures, while using the past tense for analyses and results specific to the present study. For example, in the Statistical analysis section, we revised the first sentence from “We describe the frequency and proportion ...” to “We described the frequency and proportion ...”.

2. Please ensure that every instance of “p” referring to p-values is italicized throughout the manuscript.

**Author response:** Thank you for pointing this out. We revised the manuscript to ensure that every instance of “p” referring to p-values is italicized throughout the manuscript.

## Background

3. Lines 40–47: It would be helpful to provide brief definitions and/or examples of the different PA domains (e.g., LTPA, OPA, TPA) for readers who may be less familiar with these concepts.

**Author response:** We appreciate this suggestion. To help readers who may be less familiar with domain-specific PA concepts, we added brief examples of LTPA, OPA, and TPA in the Introduction.

**Revised text:**

However, the health effects of PA may vary depending on the context in which it occurs. Leisure-time physical activity (LTPA, e.g., sports, fitness, and recreational activities) is generally reported to have clear health benefits and has been the primary basis for physical activity guidelines (PAG). By contrast, recent studies have shown that occupational physical activity (OPA, e.g., paid or voluntary work, including household chores) may increase rather than reduce the risk of cerebrovascular disease and all-cause mortality [4-6]. Active transportation is considered an effective strategy to increase PA levels for health benefits [7-9], but the relationship between transportation-related PA (TPA, e.g., walking or cycling for travel) and an increase in LTPA, as well as the health benefits based on leisure activity itself rather than the amount of PA, remains unclear [9, 10].

4. Line 48: The statement indicating that “South Korea has an exceptionally high prevalence of insufficient PA among adults” would benefit from supporting statistics and appropriate references.
5. Lines 49–50: It is unclear what criteria South Korea is being ranked on. Additional clarification would improve readability and comprehension.

**Author response:** We appreciate these helpful comments. We revised the sentence to provide supporting statistics from the WHO Global Health Observatory and to clarify the criterion used for the international ranking. Specifically, we added the age-standardized prevalence of insufficient PA among adults aged ≥18 years in South Korea and the corresponding global estimate, and clarified that the ranking was based on the same indicator among 195 countries.

**Revised text:**

South Korea has an exceptionally high prevalence of insufficient PA among adults, with the age-standardized prevalence among adults aged ≥18 years estimated at 56.5% in 2021, compared with the global estimate of 30.8%, according to the WHO Global Health Observatory [3]. Based on these WHO estimates, South Korea ranked within the top 10 among 195 countries in each year from 2016 to 2021 for this indicator.

## Methods

6. Line 63: Please define “KNHANES” at first mention and provide the appropriate reference.

**Author response:** Thank you for this comment. The abbreviation KNHANES was defined at its first mention in the Introduction, in the sentence describing the data source used for the HP2030 indicator (line 56–57). To make the description clearer in the Methods section, we revised the data source paragraph and cited appropriate references [12, 13] in the general description of KNHANES (line 69–70). Please see the revised text provided in response to Comment 8.

7. Line 64: Since the study period is reported as 2016–2021, it is unclear whether KNHANES is still ongoing. If the data collection period is completed, “aims” should likely be replaced with “aimed.”

**Author response:** We revised the paragraph to clarify that KNHANES is an ongoing national survey conducted annually by the Korea Disease Control and Prevention Agency. Accordingly, the previous wording using “aims” was removed. We retained the present tense only for general descriptions of the ongoing KNHANES and its standard procedures, while using the past tense for statements specific to the 2016–2021 data used in the present study.

8. Lines 63–70: This paragraph would benefit from further clarification. It is currently difficult to determine whether KNHANES refers to a study, a survey, or a database.

**Author response:** Thank you for this helpful comment. We revised the paragraph to clarify that KNHANES is an ongoing national survey, rather than a single study or merely a database, and that the present study used data from the 2016–2021 KNHANES. We also added that KNHANES consists of health interviews, health examinations, and nutrition surveys, and that anonymized data are publicly released through the KNHANES website for research use.

**Revised text:**

The data source for this study was the 2016–2021 KNHANES. KNHANES is an ongoing national survey conducted annually by the Korea Disease Control and Prevention Agency to monitor the health behaviors, health status, and nutritional status of the Korean population [12, 13]. The survey includes health interviews, health examinations, and nutrition surveys, and it samples the civilian, non-institutionalized population in South Korea using a complex, multistage, clustered probability sampling design. In the 2016–2021 KNHANES, each annual survey included approximately 10,000 eligible individuals selected from sampled geographic units and households, with participation rates averaging 75–78%. For all KNHANES surveys, written informed consent is obtained from all participants, and anonymized, de-identified data are made publicly available through the KNHANES website for research purposes.

9. Line 75: Please use the abbreviation “PA” consistently throughout the manuscript.

**Author response:** We reviewed the manuscript for consistent use of the abbreviation “PA” and revised “physical activity limitation” in the Methods section to “PA limitations.”

10. Line 90: There appears to be an extra character/symbol in the sentence regarding total PA time estimation.

**Author response:** Thank you for pointing this out. We removed the unnecessary parentheses in the sentence regarding total PA time estimation and revised the sentence for clarity as follows: “Total PA time was estimated by summing the times across all three domains. In this study, all PA times refer to moderate-intensity equivalent times.”

11. Lines 92–94: If this paragraph refers to GPAQ scoring, it may be preferable to avoid a line break. Otherwise, additional clarification would be appreciated.

**Author response:** Thank you for this comment. These sentences were intended to describe how PA variables were categorized for analysis, including total and domain-specific PA levels, the primary PA domain, and the contribution of each domain-specific PA to total PA. To make this clearer and improve the flow, we revised the wording of the first sentence to begin with “For analysis,” and removed the unnecessary paragraph break.

12. Lines 101–104: Please specify which health-related factors are assessed by the SRH scale.

**Author response:** SRH in KNHANES is measured using a single self-rated health question, rather than a multi-item scale assessing separate health-related factors. To avoid ambiguity, we revised the Methods section to clarify that SRH reflects respondents’ overall subjective evaluation of their health.

**Revised text:**

SRH was used as a surrogate for overall health outcomes to evaluate the association with PA patterns. In KNHANES, SRH is measured using a single question reflecting respondents’ overall subjective evaluation of their health. The question asks, “In general, would you say your health is?” with five response options ranging from “very good” to “very poor,” with “very good” and “good” grouped as “good SRH.”

13. Study variables: It may improve readability to introduce subheadings for each evaluated variable or assessment tool. Additionally, it would be useful to specify whether the measures were self-reported, interview-based, or assessed using another format.

**Author response:** We appreciate this suggestion. To improve readability, we revised the Study variables section by adding the following subheadings: “Physical activity measurement and derived variables,” “Self-rated health,” and “Sociodemographic and health-related covariates.” We also clarified the assessment format of the variables in the text, including that PA was assessed using the GPAQ, SRH was measured using a single self-rated health question, and sociodemographic and health-related covariates were obtained from the KNHANES health interview/questionnaire data, except for BMI, which was calculated from height and weight measured in the health examination.

**Revised text:**

Under “Physical activity measurement and derived variables”:
In KNHANES 2016–2021, PA levels were assessed using the GPAQ developed by WHO [14].

Under “Self-rated health”:
In KNHANES, SRH is measured using a single question reflecting respondents’ overall subjective evaluation of their health.

Under “Sociodemographic and health-related covariates”:
Sociodemographic and health-related covariates were obtained from the KNHANES health interview and questionnaire data, except for BMI, which was calculated from height and weight measured in the health examination.

14. Lines 109–112: No chronic mental health conditions appear to be included among the chronic diseases assessed. While this may reflect limitations of the existing database, acknowledging this point in the manuscript would strengthen the discussion, especially given the references to mental health in the introduction.

**Author response:** Thank you for this comment. Physician-diagnosed depression was already included in the count of chronic diseases. To clarify the handling of chronic mental health conditions, we revised the Methods section to state that chronic mental health conditions other than depression were not included because depression was the only chronic mental health condition consistently assessed among adults in the KNHANES chronic disease variables during the study period.

**Revised text:**

The number of chronic diseases was defined as the number of illnesses diagnosed by a physician among cancer, stroke, myocardial infarction or angina, hypertension, dyslipidemia, diabetes, thyroid diseases, chronic liver diseases, kidney diseases, asthma, depression, osteoarthritis or rheumatic arthritis, and gout. Chronic mental health conditions other than depression were not included in this count because depression was the only chronic mental health condition consistently assessed among adults in the KNHANES chronic disease variables during the study period.

15. Lines 121–127: This paragraph could benefit from clearer wording and restructuring to facilitate reader comprehension.

**Author response:** We revised this paragraph to improve clarity and readability by separating the descriptions of participant characteristics, PA level estimates, domain-specific PA time distributions, and PA pattern variables.

**Revised text:**

We first described the sociodemographic and health-related characteristics of the study subjects overall and according to total PA level. We then estimated the prevalence of inactivity, insufficient PA, and meeting PAG among Korean adults during 2016–2021, stratified by gender and age group (20–34, 35–49, 50–64, and 65–79 years). The distributions of total and domain-specific PA time were also estimated by gender and age group. Among those who were not inactive, we estimated the distribution of the primary PA domain and the contribution of each domain-specific PA to total PA time.

## Results

16. Tables: Please indicate the units associated with the reported values directly in the column headings (e.g., %, n).

**Author response:** We revised the table column headings so that the units or formats of the reported values can be identified directly within the tables. Specifically, in Tables 2–4, to avoid repeating “%” in each individual column heading, we added “Weighted %” as a spanning column heading over the relevant numeric columns.

17. Tables: It would also be helpful to include the mention “Note:” directly below the tables in the legends.

**Author response:** We added “Note:” before the table notes below the tables.

18. To improve readability, it is recommended to organize the Results section into clear subsections. At minimum, separate subsections could be created for participant characteristics and for each study objective. For example :
    1.1 ***Sociodemographic and health-related characteristics of participants***
    1.2 ***Evaluation of domain-specific PA patterns***
        1.2.1 *PA levels*
        1.2.2 *Prevalence of meeting PAG*
    1.3 ***Assessment of domain-specific PA patterns association with SRH for overall outcomes***
        1.3.1 *Total PA x SRH*
        1.3.2 *Domain-specific x SRH*

**Author response:** We appreciate this suggestion. To improve readability, we reorganized the Results section into clear subsections according to the main analytic components and study objectives. We added the following subsection headings: “Sociodemographic and health-related characteristics of study subjects,” “Total and domain-specific PA levels,” “Primary PA domain and contribution of each PA domain to total PA,” and “Associations between PA patterns and good SRH.”

## Discussion

19. Lines 379–385: Additional references regarding the benefits of PA on mental health would strengthen this section.

**Author response:** We added additional references to support the beneficial effects of PA, particularly LTPA, on mental health. Specifically, we added references to a population-based study on domain-specific PA and depressive symptoms and a meta-analysis of prospective cohort studies on leisure-time PA and risk of depression.

**Revised text:**

LTPA is the PA domain that is most consistently and strongly associated with mortality and health [38] and has shown beneficial effects for both physical and mental health [16, 46, 47].

The following reference was added to the reference list:

47. Guo Z, Li R, Lu S. Leisure-time physical activity and risk of depression: A dose-response meta-analysis of prospective cohort studies. Medicine (Baltimore). 2022;101(30):e29917. doi:10.1097/MD.0000000000029917. PMID: 35905243

20. Regarding the limitations associated with the GPAQ assessment tool, the authors may wish to consider discussing the SIMPAQ questionnaire as a potential avenue for future research. The SIMPAQ has demonstrated international validity and reliability in both general and mental health populations and may provide a more precise assessment of PA and sedentary behaviors. Suggested reference: Rosenbaum, S., Morell, R., Abdel-Baki, A., Ahmadpanah, M., Anilkumar, T. V., Baie, L., et al. (2020). Assessing physical activity in people with mental illness: 23-country reliability and validity of the Simple Physical Activity Questionnaire (SIMPAQ). BMC Psychiatry, 20(1), 108. <https://doi.org/10.1186/s12888-020-2473-0>

**Author response:** Thank you for this thoughtful suggestion. We carefully considered the suggested reference and the possibility of discussing the Simple Physical Activity Questionnaire (SIMPAQ) as a potential tool for future research. We recognize that SIMPAQ is a useful brief questionnaire for assessing physical activity and sedentary behavior. However, the present study focuses on domain-specific PA patterns among Korean adults using KNHANES national surveillance data and the GPAQ, particularly leisure/sports, transportation-related, and work-related PA. Because SIMPAQ does not specifically assess these domain-specific PA patterns and is not part of the KNHANES surveillance system, we felt that adding a discussion of SIMPAQ would be outside the scope and context of the present study. Therefore, we did not add this discussion to the manuscript.

We again thank the reviewer for the valuable comments and suggestions. We believe that the revisions made in response to these comments have strengthened the manuscript.
